# TCDD Inhibition of IgG1 Production in Experimental Autoimmune Encephalomyelitis (EAE) and In Vitro

**DOI:** 10.3390/antib11010004

**Published:** 2022-01-09

**Authors:** Ashleigh J. Nicaise, Amye McDonald, Erin Rushing Sears, Trell Sturgis, Barbara L. F. Kaplan

**Affiliations:** Center for Environmental Health Sciences, Department of Comparative Biomedical Sciences, College of Veterinary Medicine, Mississippi State University, Starkville, MS 39762, USA; an847@msstate.edu (A.J.N.); afmcdonald@comcast.net (A.M.); erinerushing@gmail.com (E.R.S.); qds32@msstate.edu (T.S.)

**Keywords:** aryl hydrocarbon receptor, TCDD, experimental autoimmune encephalomyelitis

## Abstract

The environmental contaminant 2,3,7,8-tetrachlorodibenzo-para-dioxin (TCDD) is a ligand for the aryl hydrocarbon receptor (AhR). TCDD is well-characterized to produce immunotoxicity, including suppression of antibody production. Previously we showed that TCDD inhibited myelin oligodendrocyte glycoprotein (MOG) peptide-specific IgG and attenuated disease in experimental autoimmune encephalomyelitis (EAE) model in mice. Thus, the purpose of this study was to characterize the effects of TCDD on IgG subclasses in EAE and in vitro and assess effects in B cells derived from various tissues. TCDD modestly suppressed intracellular IgG expression in splenocytes (SPLC), but not bone marrow (BM) or lymph node (LN) cells. To further understand TCDD’s effects on IgG, we utilized LPS and LPS + IL-4 in vitro to stimulate IgG3 and IgG1 production, respectively. TCDD preferentially suppressed IgG1+ cell surface expression, especially in SPLC. However, TCDD was able to suppress IgG1 and IgG3 secretion from SPLC and B cells, but not BM cells. Lastly, we revisited the EAE model and determined that TCDD suppressed MOG-specific IgG1 production. Together these data show that the IgG1 subclass of IgG is a sensitive target of suppression by TCDD. Part of the pathophysiology of EAE involves production of pathogenic antibodies that can recruit cytolytic cells to destroy MOG-expressing cells that comprise myelin, so inhibition of IgG1 likely contributes to TCDD’s EAE disease attenuation.

## 1. Introduction

TCDD is an environmental contaminant that exhibits toxicity, especially in the liver, skin and immune system via the aryl hydrocarbon receptor (AhR) [1]. The mechanisms by which TCDD suppresses B cell function involve inhibition of activation and differentiation into plasma cells [2]. For instance, in mouse B cells, TCDD-mediated suppression of IgM occurs in an AhR-dependent manner [3] and involves inhibition of several transcriptional regulators critical for antibody production [4,5,6]. TCDD also suppresses IgG antibody production [7,8,9,10,11,12,13].

In humans, there is evidence that exposure to TCDD has resulted in reduced circulating IgG levels. Following an industrial accident in Italy in which people were exposed to dioxin, plasma IgG levels were inversely proportional with blood TCDD levels, and the IgG levels of those exposed to TCDD were lower as compared to a non-exposed control cohort [14]. In a German cohort in which body burden of TCDD and immune endpoints were evaluated, again there was a slight but statistically significant decrease in plasma IgG1 with increased blood levels of TCDD [15]. The inverse relationship between TCDD levels and IgG was also noted in New Zealand and Korean cohorts [16,17].

Experimental autoimmune encephalomyelitis (EAE) is an autoimmune disease model of multiple sclerosis that can be induced in mice using the myelin oligodendrocyte glycoprotein (MOG) peptide. TCDD has been shown to suppress EAE disease through inhibition of TH1 and TH17 responses [18]. Previously we showed that part of the mechanism by which TCDD suppressed EAE clinical disease involved inhibition of intracellular IgG in CD19- cells in the spleen and spinal cord and suppression of total and MOG-specific serum IgG [11]. The disease process in EAE involves production of pathogenic antibodies that can recruit cytolytic cells to destroy MOG-expressing cells that comprise myelin. For instance, some subclasses of IgG effectively activate complement-mediated lysis and antibody-dependent cell-mediated cytotoxicity (ADCC) [19], which could be involved in destruction of myelin and/or myelin-producing cells [20]. Thus, the purpose of these studies was to further characterize the effects of TCDD on IgG antibody production. Toward that end, we used the EAE model in vivo and LPS- or LPS + IL-4-stimulated cells in vitro. Our data show that IgG1 is a sensitive target of suppression by TCDD, which might lead to subsequent compromised antibody-dependent immune defenses. Thus, these studies provide further information on the mechanism by which TCDD suppresses immune function. While TCDD could not be developed as an immunosuppressive therapeutic due to its systemic toxicity, these data suggest that other non-toxic AhR ligands might possess efficacy for the treatment of immune-mediated diseases.

## 2. Materials and Methods

Animals. Adult female C57BL/6 mice were obtained from Envigo (Indianapolis, IN, USA). Splenocytes or BM cells were obtained from naïve mice for in vitro cultures. BM cells were obtained by flushing femurs with 1X phosphate-buffered saline (PBS). EAE was induced using 100 µg myelin oligodendrocyte glycoprotein peptide (MOG; amino acids 35–55, MEVGWYRSPFSRVVHLYRNGK) emulsified in Complete Freund’s Adjuvant supplemented with heat-killed *Mycobacterium tuberculosis* (total 5 mg/mL) as a subcutaneous injection in four flanks on day 0. As previously established, we omit pertussis toxin to induce a milder disease state more consistent with autoimmune disease onset [21]. Indeed, in this study EAE disease was mild but TCDD did not produce clinical signs at all (Supplemental Figure S1), similar to what we observed in our previous publications [11,21]. TCDD (2.5 µg/kg/day; Accustandard, New Haven, CT, USA) was administered in corn oil (CO) vehicle on days 1–12 days via oral gavage, and mice were euthanized at day 18. Four groups were used: saline-injected with CO vehicle (SAL/CO), saline-injected with TCDD (SAL/TCDD), MOG in CFA-injected with CO vehicle (EAE/CO) and MOG in CFA-injected with TCDD (EAE/TCDD). Cells from spleen, BM and draining lymph nodes (axillary and inguinal LN) were harvested. All work with animal subjects was conducted in accordance with the Mississippi State University Institutional Animal Care and Use Committee (protocol numbers (17-427 and 20-219 to BLFK).

Reagents. MOG peptide was obtained from Biosynthesis (Lewisville, TX, USA). Heat-killed *Mycobacterium tuberculosis* H37Ra (HKMT) was obtained from Difco/BD Biosciences (Detroit, MI, USA), and Complete Freund’s Adjuvant (CFA) was obtained from Sigma (St. Louis, MO, USA). LPS (*E. coli* 055:B5) was obtained from Sigma. Recombinant mouse IL-4 was obtained from R and D Systems (Minneapolis, MO, USA). All other chemicals/reagents were obtained from Fisher Scientific unless otherwise noted.

Antibodies. All antibodies were obtained from Biolegend (San Diego, CA, USA) unless otherwise indicated. Antibodies for immunofluorescence analysis were CD19-PECy7 (clone 6D5), CD5-Brilliant Violet (BV) 421 (clone 53-73), B220-BV785 (clone RA3-4B2), IgG1-APC (clone RMG1-1) and IgG3-Dylight488 (polyclonal; Novus Biologicals, Centennial, CO, USA). Antibodies for the IgG1 ELISAs were purified and biotinylated anti-mouse IgG1 (both clone RMG1-1). IgG3 ELISA antibodies were obtained from BD Biosciences (San Jose, CA, USA) and included the purified (clone R2-38) and biotinylated forms (clone R40:82).

Cell cultures. Splenocytes (SPLC) or bone marrow (BM) cells were obtained from naïve mice and single cell suspensions were generated. B cells were purified from splenocytes using the naïve B cell isolation kit (Stem Cell, Cambridge, MA, USA). SPLC or B cells were seeded in 48 well plates at 2 × 10^6^ cells/mL in 1 mL complete media (1× RPMI containing 5% bovine calf serum, 1% penicillin-streptomycin and 50 µM 2-mercaptoethanol). BM cell yields were typically lower than SPLC or B cells, so BM cells were seeded in the range of 0.8–2 × 10^6^ cells per ml for fewer days of culture. Cells were treated with vehicle (0.01% dimethylsulfoxide, DMSO) or TCDD (30 nM) for 30 min before receiving stimulation. Cells were stimulated with LPS (5 µg/mL) with or without recombinant mouse IL-4 (10 ng/mL) for up to 4 days at 37 °C. No treatments, stimulation or media were removed or replenished for the duration of the culture period.

ELISAs. For MOG-specific IgG1 ELISA, plates were coated with 100 µg/mL MOG peptide in 1X PBS overnight at 4 °C. For other IgG ELISAs, purified anti-mouse IgG1 or IgG3 antibodies were diluted in sodium bicarbonate (IgG1) or 1X PBS (IgG3) overnight at 4 °C. Plates were washed three times with 0.05% Tween-20 in 1X PBS followed by three times in deionized (DI) water. Plates were blocked with 3% bovine serum albumin (BSA) in 1X PBS for at least 1 h at room temperature (RT). For MOG-specific IgG1, 25 µL of serum samples isolated from the blood of EAE or control animals was added to the plate for at least 1 h at RT. For IgG1 and IgG3, supernatants from in vitro cultures were added to the plates for at least 1 h at RT. After another wash cycle, IgG1 biotinylated secondary antibody was used for MOG-specific IgG1 and IgG1, and IgG3 biotinylated secondary antibody was used for IgG3. Again, after washing, plates were assayed for absorbance at 450 nm following subsequent addition of horseradish peroxidase avidin, tetramethylbenzidine substrate and 2N H2SO4 with wash steps in between avidin and substrate. ELISA data were normalized using counts from cells gathered at the same time as the supernatants using the NovoCyte flow cytometer (ACEA/Agilent, Santa Clara, CA, USA) gated on forward and side scatter characteristics (FSC, SSC) typical of leukocytes.

Immunofluorescence analysis. Cells were harvested and washed once in 1X PBS. Cells were then stained with near-IR fixable viability dye (Biolegend) for 20 min in 1X PBS at RT in the dark. After a wash in 1X PBS, cells were incubated with FcBlock (BD Biosciences) for 10 min then incubated with antibodies for CD19, B220, CD5, IgG1 and IgG3 for 30 min at RT in the dark. Cells were then fixed with Cytofix (BD Biosciences), washed, and resuspended in flow cytometry buffer (1% BSA in 1X Hank’s Buffered Saline Solution) for analysis. Cells were analyzed on a NovoCyte, and data were analyzed using the NovoExpress software version 1.4.1 (Agilent).

Real time RT-qPCR. Total RNA was isolated using an RNeasy kit (Qiagen, Germantown, MD, USA). After quantifying the RNA using a Nanodrop, cDNA was synthesized (cDNA Archive Kit, Applied Biosystems/ThermoFisher, Waltham, MA, USA) and used to assess gene expression by Taqman PCR (*Cyp1a1* primer probe Mm00487218_m1; *Aicda* primer probe Mm1184115_m1). Data were normalized to the untreated (Untx) sample on day 2 using the ΔΔCt method [22].

Statistical analysis. Data are presented as the mean ± SD. One-way ANOVA was performed on data in which a single dependent variable was quantified while two-way ANOVA was performed on data with two dependent variables. Grubb’s test was used at *p* < 0.05 to identify outliers. Percent and other non-Gaussian data were transformed prior to ANOVA or a non-parametric Friedman’s ANOVA followed by Dunn’s post hoc test was used. Other differences between groups were detected using Dunnett’s post hoc test following one-way AVOVA and Tukey’s multiple comparison test following two-way ANOVA.

## 3. Results

### 3.1. TCDD Modestly Suppressed the Percent of Intracellular IgG in CD19-, B220- and CD5- Cells in EAE

**Figure 1:**
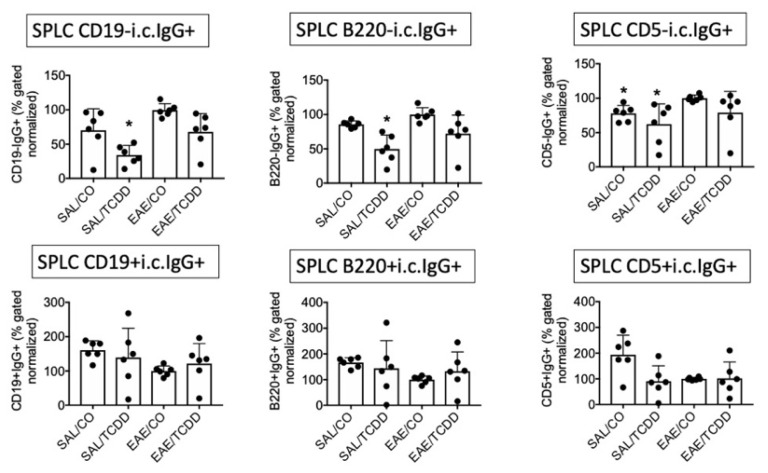
TCDD modestly suppressed i.c. IgG in SPLC in EAE. EAE was induced in mice using active immunization with MOG peptide on day 0. TCDD (2.5 µg/kg/day) in corn oil (CO) was administered via oral gavage for 12 days for a cumulative dosage of 30 µg/kg. At day 18, mice were sacrificed and SPLC were stained for extracellular B cell markers and i.c. IgG. Positive or negative B cell markers plus i.c. IgG double positive percentages were normalized in each of two experiments to the average of the EAE/CO groups (*n* = 3 per experiment) to which all other groups were compared in that experiment (total *n* = 6). Graphs represent average ± SD of normalized values from the two separate experiments. * *p* < 0.05 as compared to EAE/CO using Friedman’s ANOVA and Dunn’s post hoc test. i.c., intracellular; SPLC, splenocytes; SAL, saline.

Previously we showed that, following 12 days of oral administration, TCDD inhibited MOG-specific IgG, inhibited intracellular (i.c.) IgG in spleen and spinal cord, and decreased EAE clinical disease at 18 days [11]. To better characterize the IgG response in EAE, we evaluated i.c. IgG in various anatomic locations with several markers. We use i.c. IgG to indicate cells that are class-switched and activated in EAE [11]. As noted in our earlier work, TCDD modestly suppressed i.c. IgG in SPLC at end-stage disease, which was predominantly noted in the CD19- population but not in the CD19+ population. In these two experiments we detected a similar trend in that the i.c. IgG in the CD19- population in EAE/TCDD mice was lower than in EAE/CO mice, although this was not statistically significant. However, we did detect a significant suppression by SAL/TCDD in the CD19-i.c. IgG+, B220-i.c. IgG+ and CD5-i.c. IgG+ populations (Figure 1).

**Figure 2:**
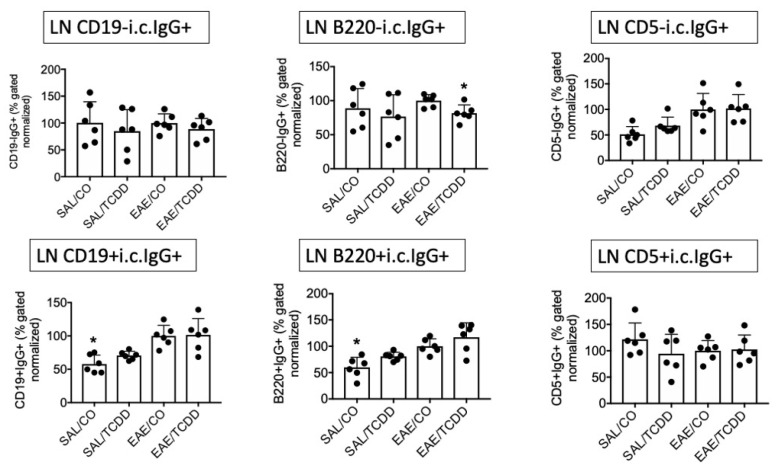
TCDD effect on i.c. IgG in LN in EAE. Samples were obtained as in Figure 1, but from LN. * *p* < 0.05 as compared to EAE/CO using Friedman’s ANOVA and Dunn’s post hoc test. i.c., intracellular; LN, lymph nodes.

**Figure 3:**
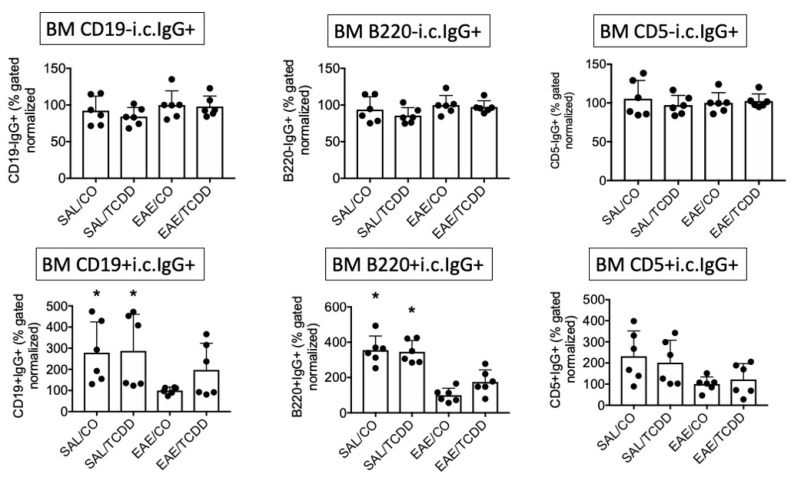
TCDD modestly reversed EAE-induced downregulation of i.c. IgG in BM in EAE. Samples were obtained as in Figure 1, but from BM. * *p* < 0.05 as compared to EAE/CO using Friedman’s ANOVA and Dunn’s post hoc test. i.c., intracellular; BM, bone marrow.

As a comparison, we show here that the B220-i.c. IgG+ and CD5-i.c. IgG+ populations are similarly affected by TCDD, although again, effects were modest. There were few differences in the percentage of i.c. IgG from B cells derived from LN or BM at end-stage disease, although there was a trend toward reversing the EAE-induced reduction in i.c. IgG on CD19+ and B220+ B cells in the BM (Figure 2 and Figure 3).

**Figure 4:**
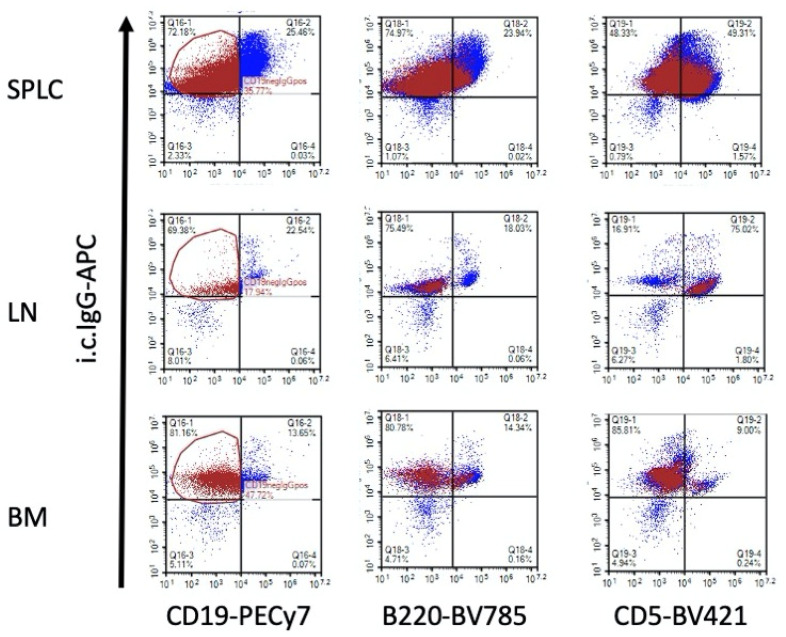
Overlap between CD19-i.c. IgG+, B220-i.c. IgG+ and CD5-i.c. IgG+ populations. Using one of the SAL/CO samples, CD19-i.c. IgG+ cells were identified in the dot plot as red so that overlapping populations could be detected across gates and tissues. Data from one of the SAL/CO samples of one of the two experiments are representative.

It was interesting to note that the CD19-i.c. IgG+ population had significant overlap with the B220-i.c. IgG+ in SPLC, LN and BM (Figure 4) suggesting these are the same cell population. On the other hand, there was also overlap between the CD19-i.c. IgG+ with CD5+i.c. IgG+ in SPLC and especially in LN (Figure 4, middle right).

### 3.2. TCDD Suppressed the Percent of IgG1, but Not IgG3, Expressed on the Cell Surface

**Figure 5:**
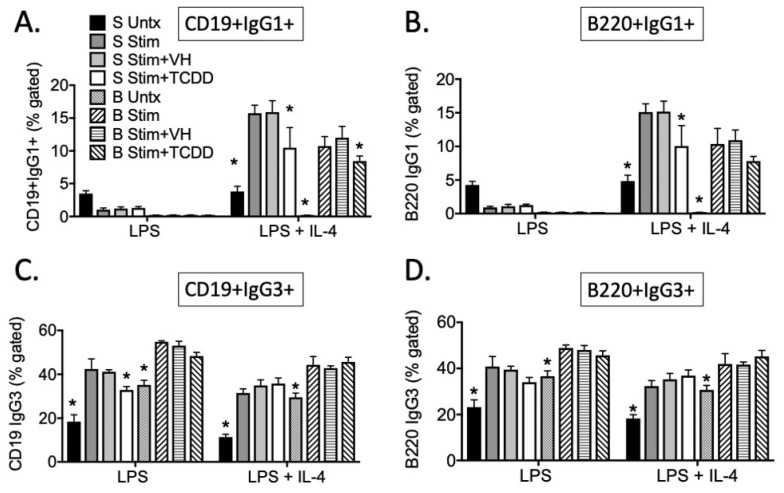
TCDD inhibited the percentage of CD19+IgG1+ cells. SPLC or B cells were treated with VH (0.01% DMSO) or TCDD (30 nM) for 30 min then were stimulated with LPS (5 µg/mL) alone or with IL-4 (10 ng/mL). Cells were incubated for 4 days then stained for CD19, B220, IgG1 and IgG3. Cells were gated on live single lymphocytes. (**A**) CD19+IgG1+; (**B**) B220+IgG1+; (**C**) CD19+IgG3+; (**D**) B220+IgG3+. Bars represent mean ± SD from triplicate samples. S, SPLC; B, B cells; Stim, LPS or LPS + IL-4; VH, vehicle; Untx, untreated. * *p* < 0.05 as compared to respective Stim + VH control within cell type. Experiments were repeated at least twice.

In order to understand some of the mechanisms by which TCDD inhibited IgG, we utilized LPS or LPS + IL-4 to stimulate B cells in vitro. LPS preferentially stimulates IgG3 while the addition of IL-4 can induce both IgG1 and IgE [20], although here we only focused on cell surface expression of IgG subclasses (Figure 5).

**Figure 6:**
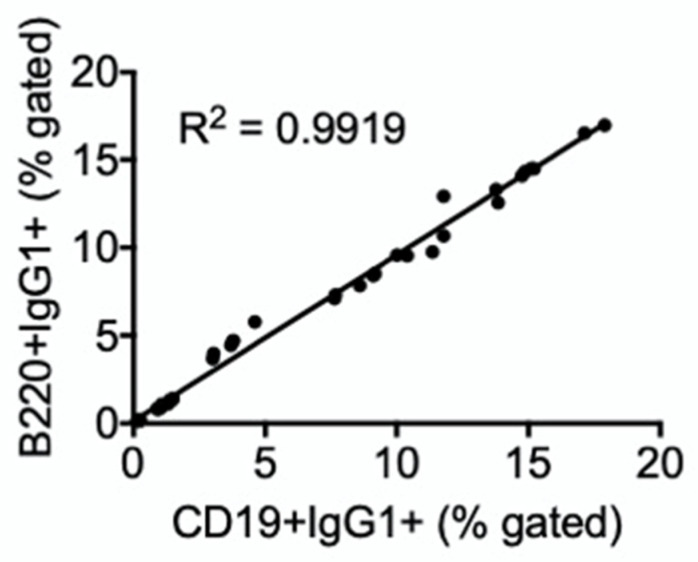
Percent of CD19+IgG1+ and B220+IgG1+ cells were correlated. Percent CD19+IgG1+ and B220+IgG1+, regardless of treatment, were plotted. Data were obtained from one of the representative in vitro experiments in which IgG1 was evaluated on CD19+ and B220+ cells. The positive correlation regression line is significant at *p* < 0.0001.

Treatment of cells with TCDD suppressed the percentage of IgG1+ cells regardless of whether they were co-stained with CD19 or B220 (Figure 5A,B). However, TCDD did not affect the percentage of cells expressing IgG3 (Figure 5C,D). It was interesting that the percentage of cells that expressed CD19 and B220 was highly correlated; for instance, there was a direct positive correlation between percent CD19+IgG1+ and B220+IgG1+ cells in SPLC regardless of treatment (Figure 6).

**Figure 7:**
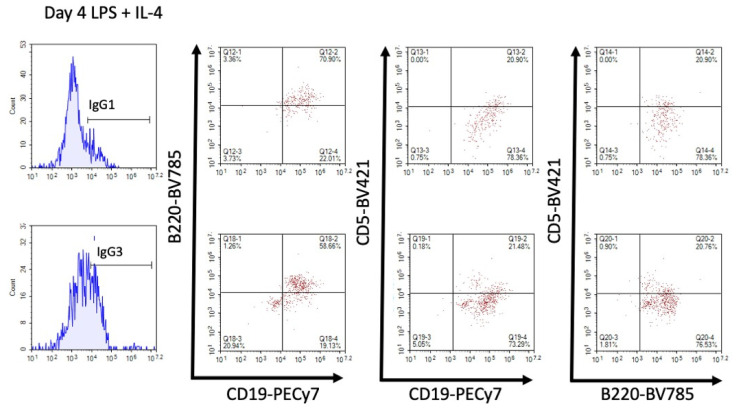
CD19 and B220 were highly co-expressed. Using LPS + IL-4-stimulated SPLC from day 4, cells were pre-gated for total IgG1 or IgG3 from the live single lymphocyte population then assessed for CD19, B220 and CD5. Top, IgG1; bottom, IgG3. Data from one of the LPS + IL-4 samples of one of at least two experiments are representative.

Further investigation into the cell types producing the antibodies revealed that CD19 and B220 were usually co-expressed in the LPS + IL-4 stimulated group at day 4 (Figure 7). A smaller percentage of cells co-expressed CD5 with either CD19 or B220.

**Figure 8:**
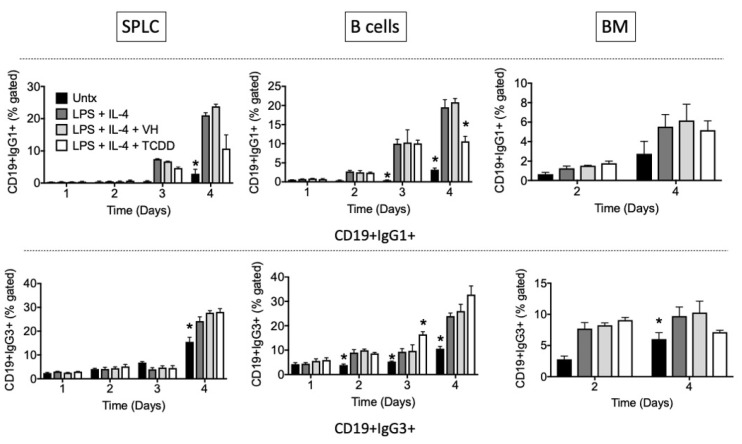
TCDD inhibited the percentage of CD19+IgG1+ cells in B cells at day 4. SPLC, B cells or BM cells were treated with VH (0.01% DMSO) or TCDD (30 nM) for 30 min then were stimulated with LPS + IL-4 (5 µg/mL + 10 ng/mL). Cells were incubated for 1, 2, 3 or 4 days then stained for CD19, B220, CD5 IgG1 and IgG3 (top, IgG1; bottom, IgG3; CD19 shown only). Cells were gated on live single lymphocytes. Bars represent mean ± SD from triplicate samples. Untx, untreated; VH, vehicle. * *p* < 0.05 as compared to respective LPS + IL-4 + VH control within day. Experiments were repeated at least twice.

**Figure 9:**
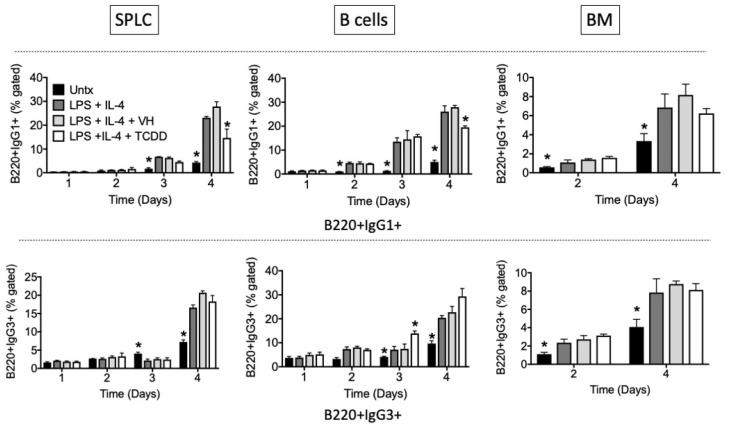
TCDD inhibited the percentage of B220+IgG1+ cells in SPLC and B cells at day 4. SPLC, B cells or BM cells were treated with VH (0.01% DMSO) or TCDD (30 nM) for 30 min then were stimulated with LPS + IL-4 (5 µg/mL + 10 ng/mL). Cells were incubated for 1, 2, 3 or 4 days then stained for CD19, B220, CD5, IgG1 and IgG3 (top, IgG1; bottom, IgG3; B220 shown only). Cells were gated on live single lymphocytes. Bars represent mean ± SD from triplicate samples. Untx, untreated; VH, vehicle. * *p* < 0.05 as compared to respective LPS + IL-4 + VH control within day. Experiments were repeated at least twice.

**Figure 10:**
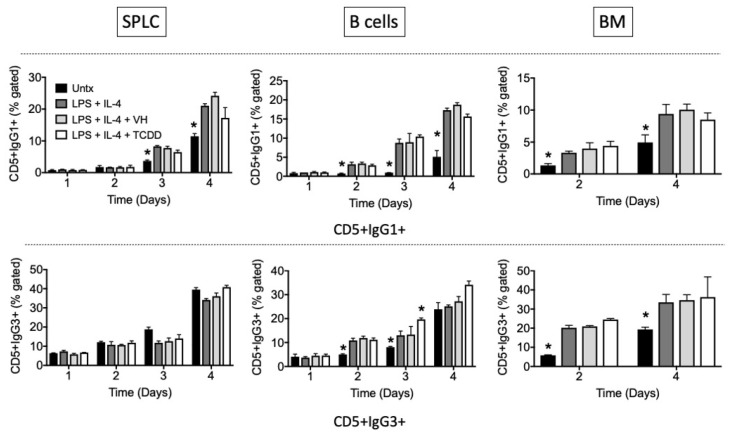
TCDD did not inhibit the percentage of CD5+IgG1+ or CD5+IgG3+ cells. SPLC, B cells or BM cells were treated with VH (0.01% DMSO) or TCDD (30 nM) for 30 min then were stimulated with LPS + IL-4 (5 µg/mL + 10 ng/mL). Cells were incubated for 1, 2, 3 or 4 days then stained for CD19, B220, CD5 IgG1 and IgG3 (top, IgG1; bottom, IgG3; CD5 shown only). Cells were gated on live single lymphocytes. Bars represent mean ± SD from triplicate samples. Untx, untreated; VH, vehicle. * *p* < 0.05 as compared to respective LPS + IL-4 + VH control within day. Experiments were repeated at least twice.

We next examined TCDD’s effects over a time course of IgG1 or IgG3 induction in total SPLC, purified B cells, or BM cells. The results confirm those seen on day 4 in which TCDD’s predominant effect is inhibition of IgG1 cell surface expression with little effect on IgG3, except for stimulation of an IgG3+ population at day 3 in B cells (Figure 8, Figure 9 and Figure 10).

**Figure 11:**
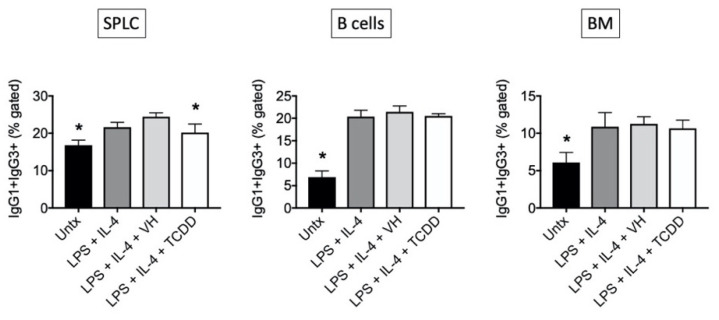
TCDD inhibited percentage of cells co-expressing IgG1 and IgG3. SPLC, B cells or BM cells were treated with VH (0.01% DMSO) or TCDD (30 nM) for 30 min then were stimulated with LPS + IL-4 (5 µg/mL + 10 ng/mL). Cells were incubated for 1, 2, 3 or 4 days then stained for CD19, B220, CD5 IgG1 and IgG3. Cells were gated on live single lymphocytes then the double positive population for IgG1 and IgG3 was calculated on day 4 only. Bars represent mean ± SD from triplicate samples. Untx, untreated; VH, vehicle. * *p* < 0.05 as compared to respective LPS + IL-4 + VH control within cell type. Data were obtained from one of the representative in vitro experiments in which IgG1 and IgG3 were evaluated on CD19+ and B220+ cells.

Again, similar patterns were observed in CD19+ and B220+ cells (Figure 8 and Figure 9), and there is a greater effect of TCDD on IgG1 in SPLC and B cells as opposed to BM cells (Figure 8). CD5+ B cells were less sensitive to suppression of IgG1 by TCDD regardless of tissue of origin (Figure 10). Disregarding any B cell surface markers, TCDD suppressed the percentage of extracellular IgG1+IgG3+ double positive cells in SPLC only (Figure 11).

**Figure 12:**
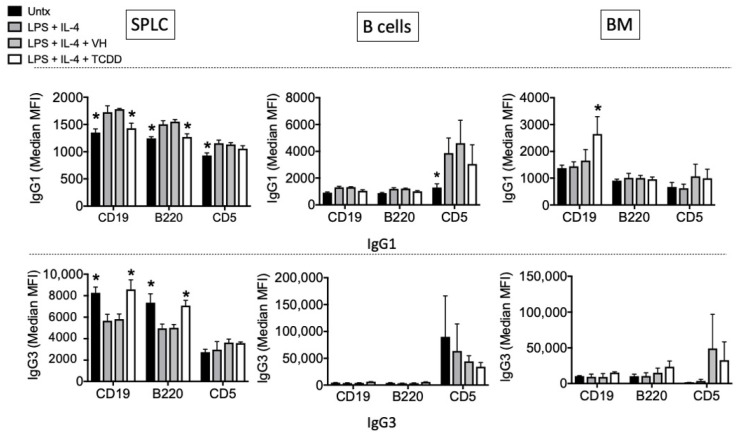
TCDD inhibited the MFI of IgG1 in SPLC. SPLC, B cells or BM cells were treated with VH (0.01% DMSO) or TCDD (30 nM) for 30 min then were stimulated with LPS + IL-4 (5 µg/mL + 10 ng/mL). Cells were incubated for 1, 2, 3 or 4 days then stained for CD19, B220, CD5 IgG1 and IgG3 (top, IgG1; bottom, IgG3; day 4 shown only). Cells were gated on live single lymphocytes. Bars represent mean ± SD from triplicate samples. Untx, untreated; VH, vehicle. * *p* < 0.05 as compared to respective LPS + IL-4 + VH control within B cell marker. Data were obtained from one of the representative in vitro experiments in which IgG1 and IgG3 were evaluated on CD19+ and B220+ cells.

An examination of the expression of IgG1 and IgG3 using mean fluorescence intensity (MFI) on the various B cell subsets at day 4 showed that IgG1 was more sensitive to suppression by TCDD, especially in SPLC (Figure 12). There was no suppression of the percentage of IgG3-expressing cells by TCDD in any tissue type at day 4 post LPS + IL-4, and again expression percentage of IgG1 or IgG3 on CD5+ cells was not affected by TCDD in any tissue, although CD5+ cells showed higher expression of both antibodies on the cell surface in the purified B cell population.

### 3.3. TCDD Suppressed IgG1 and IgG3 Antibody Secretion

**Figure 13:**
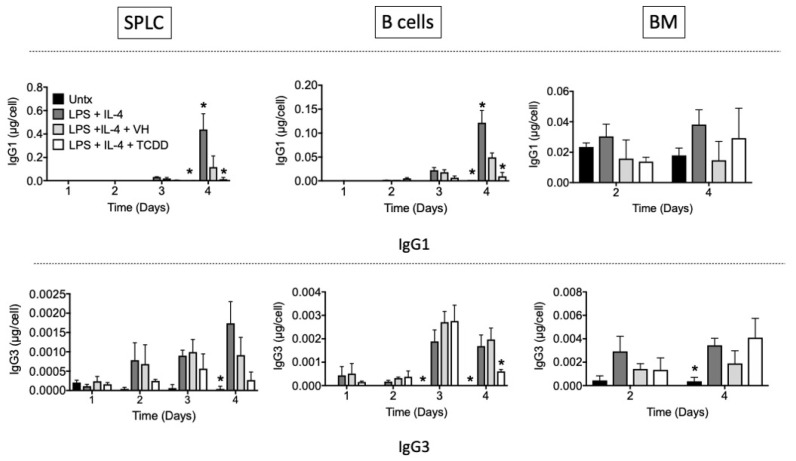
TCDD inhibited IgG1 and IgG3 antibody secretion. SPLC, B cells or BM cells were treated with VH (0.01% DMSO) or TCDD (30 nM) for 30 min then were stimulated with LPS + IL-4 (5 µg/mL + 10 ng/mL). Cells were incubated for 1, 2, 3 or 4 days then supernatants were collected and assayed by ELISA (top, IgG1; bottom, IgG3). Antibody levels were quantified as a concentration compared to a standard curve then normalized based on cell counts obtained from the Novocyte flow cytometer on each day. Bars represent mean ± SD from triplicate samples. Untx, untreated; VH, vehicle. * *p* < 0.05 as compared to respective LPS + IL-4 + VH control within day.

Given the differential effect of TCDD on IgG1 and IgG3 cell surface expression, we assessed whether antibody secretion would be affected. IgG1 antibody secretion was not readily detected until day 4 after LPS + IL-4 stimulation. Although the 0.01% DMSO vehicle produced significant suppression alone, TCDD further suppressed IgG1 as compared to the vehicle in SPLC and B cells (Figure 13). IgG3 was also induced by LPS + IL-4 by day 4 although the amount of IgG3 secretion was far lower than IgG1 in cells derived from all tissues. TCDD suppressed IgG3 secretion at day 4, but there was no effect of TCDD on IgG1 or IgG3 secretion from cells derived from BM.

### 3.4. TCDD Induced Cyp1a1 Gene Expression with No Effect on Aicda

**Figure 14:**
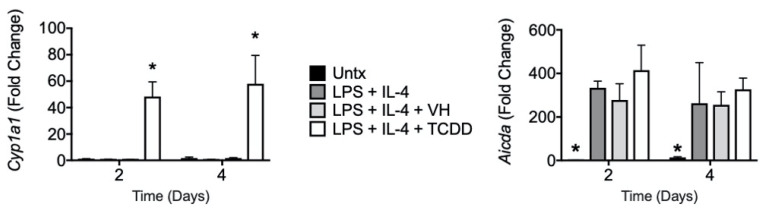
Effect of TCDD on gene expression. SPLC were treated with VH (0.01% DMSO) or TCDD (30 nM) for 30 min then were stimulated with LPS + IL-4 (5 µg/mL + 10 ng/mL). Cells were incubated for 2 or 4 days then total RNA was isolated. RT-qPCR was performed for *Cyp1a1* and *Aicda*. Bars represent mean ± SD from triplicate samples. Untx, untreated; VH, vehicle. * *p* < 0.05 as compared to respective LPS + IL-4 + VH control within day.

It has been reported that one possible mechanism for AhR ligand-mediated suppression of antibody production is inhibition of the gene encoding the class switch recombination enzyme, activation-induced cytidine deaminase (AID), by AhR [13]. While TCDD robustly induced *Cyp1a1* as expected, there was no effect on LPS + IL-4-stimulated *Aicda* expression (Figure 14).

### 3.5. TCDD Inhibited MOG-Specific IgG1 in EAE Mice

**Figure 15:**
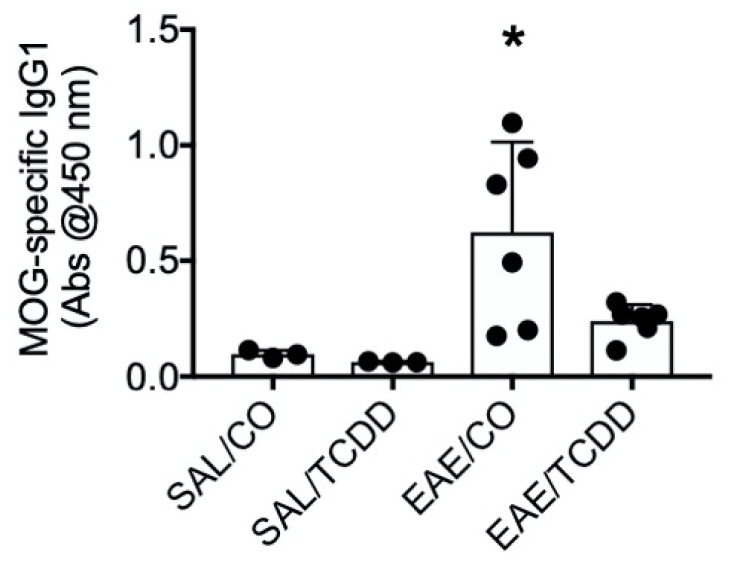
TCDD inhibited MOG-specific IgG1 in EAE. Mice were immunized with MOG peptide in CFA on day 0. Mice received CO or 2.5 µg TCDD/kg/day via oral gavage for 12 days. On day 18, blood was collected, and serum was isolated from individual mice. Serum was assayed in an ELISA. Bars represent mean ± SD from separate mice (*n* = 3 or 6). Results are representative of 3 separate experiments. SAL, saline; CO, corn oil. * *p* < 0.05 as compared to EAE/CO.

Given the larger role for IgG1 as compared to IgG3 and its enhanced susceptibility to suppression by TCDD, we again used the EAE model to determine whether IgG1 was affected by TCDD in vivo. We obtained serum from the blood of mice undergoing EAE and treated with TCDD and noted that TCDD suppressed MOG-specific IgG1 (Figure 15).

## 4. Discussion

The studies herein extensively characterize the effects of TCDD in B cells derived from various anatomic locations in EAE in vivo and in response to LPS + IL-4 in vitro. In EAE we found that TCDD modestly suppressed intracellular IgG in CD19-, B220- and CD5- SPLC at end-stage disease (i.e., day 18 in our model [11,21]) with minor effects on antibody expression in LN or BM. While we did not obtain statistically significant suppression of the CD19-i.c. IgG+ population in EAE in SPLC as we did previously [11], the trend is consistent. This could be due to the fact that the clinical outcome in our model is slower to develop but likely similar to disease onset in humans. One challenge that does result from this is that we get variability across animals with some of our in vivo results, but we believe the slower onset EAE model provides important information about TCDD’s effects in a model of early disease [21].

We also determined that there was significant overlap in the CD19-i.c. IgG+ and B220-i.c. IgG+ populations, strongly suggesting these are the same cells. Some of the CD19- and B220- cells, however, could be CD5+i.c. IgG+ cells, especially in the SPLC and LN. The lack of effect of TCDD in LN or BM is likely due to timing; we suspect day 18 is past the peak time to measure MOG-induced IgG in LN or BM. The lack of a suppressive effect by TCDD on IgG in the BM could also be due the fact that long-lived plasma cells are more important in the BM [23] and our measure of intracellular IgG is more indicative of a plasmablast [24]. The data also suggest, albeit the effect is modest, that EAE reduced the percentage of intracellular IgG-expressing B cells in BM, which might indicate they trafficked to the spleen or sites of inflammation such as the spinal cord and brain, and that TCDD might retain them in the BM as part of the mechanism to reduce neuroinflammation and EAE clinical signs.

We undertook in vitro studies in an attempt to further understand the effect of TCDD on IgG and it has been established that using LPS versus LPS + IL-4 preferentially induces IgG1 as compared to IgG3 [25]. Effects of TCDD on IgG1 production have been established by others [8,9,10,12], but fewer studies have examined IgG3 [13]. In humans it was also noted that increased body burden of TCDD was associated with decreased plasma IgG, which was mainly due to a decrease in IgG1 levels [15]. Moreover, most of the previous studies have primarily measured antibody secretion in culture supernatants or in serum, and the present studies allowed us to evaluate which B cells were sensitive to suppression by TCDD and whether that correlated to effects on secretion. Interestingly, we did not detect major differences in TCDD sensitivity between SPLC and B cells, with the exception of IgG1 or IgG3 MFI. We were surprised initially that the percentage and expression of IgG3 was not suppressed (and in fact on day 3 there was induction of an IgG3+ population), so it was important to evaluate whether TCDD would inhibit IgG3 secretion, which it did. The fact that TCDD did inhibit secretion of both IgG1 and IgG3 is consistent with the reported regulation of the gene encoding the class switch recombination enzyme, AID, by AhR [13], although we did not detect any effect of TCDD on *Aicda* gene expression. There is another study in which the AhR ligand, ITE, inhibited antibody production, but did not alter *Aicda* gene expression [26], suggesting additional mechanisms must be involved in TCDD-mediation inhibition of IgG1 class switch and/or secretion. It was interesting to note that in the same study in which the role of AhR in regulation of AID was noted, the LPS-induced percentage of IgG3+B220+ was not suppressed by TCDD, consistent with our observations here (although they did not measure IgG3 secretion) [13]. The authors did note however that LPS + anti IgD dextran-induced percentage of IgG3+B220+ was suppressed by TCDD and they attributed the differential sensitivity to TCDD to the fact that LPS alone did not induce AhR protein expression, but LPS + anti IgD dextran did, rendering only the LPS + anti IgD dextran-treated cells sensitive to suppression by TCDD [13].

While we did not use an AhR antagonist (i.e., CH223191) in these studies, there is other evidence that suppression of antibody production is AhR-dependent. Specifically focusing on IgG1, Yoshida and colleagues used the AhR ligand, ITE, and showed that it inhibited CD40 ligand plus IL-4-stimulated IgG1 production in mouse splenic B cells, and that CH223191 alone stimulated IgG1 production, suggesting a role for AhR [26]. Another study showed that CH223191 reversed TCDD-induced suppression of LPS-stimulated IgG2b and IgA production in a mouse B cell line [27]. Further, they demonstrate that in AhR knockdown cells, TCDD did not suppress IgG2b or IgA antibody production [27].

Consistently we observed that antibody staining on CD19+ and B220+ cells was similar, and a regression analysis confirmed a strong positive correlation between CD19+IgG1+ and B220+IgG1+ cells in SPLC regardless of treatment. This was likely due to the fact that IgG1- or IgG3-expressing cells came predominantly from cells that co-expressed CD19 and B220. The co-expression of intermediate levels of CD19 and B220 in SPLC in part define a dividing plasmablast [28], which is likely the B cell stage induced by LPS + IL-4 at 4 days. There were smaller percentages of CD5+ cells co-expressing CD19 or B220, but this was expected as CD5 in part defines a regulatory B cell population [29,30]. However, the CD5+ cells also produced IgG1 and IgG3 in response to LPS + IL-4, so these CD5+ cells likely also play a role in immune defense [30]. Recently in human B cells, it was observed that TCDD modestly increased IgG in CD5- B cells in response to CD40L + IL-2 and IL-21 for 7 days [31].

As part of our assessment of the co-expressed molecules in response to LPS + IL-4, we also determined if TCDD altered IgG1+IgG3+ cells and found that TCDD only produced significant suppression of this double positive population in SPLC. In SPLC the percent of IgG1+ cells was consistently inhibited with little effect on the percent of IgG3+ cells by TCDD regardless of the B cell marker co-expression. In B cells, although the percent of IgG1+ cells was consistently inhibited, there were slight increases in the percent of IgG3+ cells by TCDD so when examined together, there was no effect of TCDD on IgG1+IgG3+ cells. These data are consistent with recent observations showing in human B cells ~18–44% of individual B cells expressed the genes for two Ig classes allowing for a single B cell to express multiple distinct Igs [32].

Upon noting the preferential effect of TCDD on IgG1 in vitro, we revisited the EAE model and demonstrated that TCDD suppressed circulating MOG-specific IgG1. We previously demonstrated that TCDD inhibited MOG-specific IgG [11], so we now know that part of the IgG pool that was affected by TCDD was IgG1. IgG1 effectively activates complement-mediated lysis and ADCC [19], which could be involved in destruction of myelin and/or myelin-producing cells [20]. The specific mechanisms for antibodies in demyelination are not totally understood, but IgG1-producing clones have been generated from MS patients and the amount of demyelination correlated positively with the complement C9 protein following disease induction with anti-MOG IgG1 antibodies [33]. On the other hand, other components of complement were not required for demyelination and/or EAE disease such as complement protein C3 [34] or the membrane attack complex [35]. These results suggest that the major mechanism for demyelination is ADCC, perhaps from macrophages [33]. A more recent study also suggested that antibody-mediated opsonization of autoantigen might contribute to demyelination [36]. Moreover, other IgG subclasses might also be involved in demyelination, including IgG2a [33], so the mechanism by which TCDD suppressed EAE and demyelination [21] could also include suppression of MOG-specific IgG2a.

Together these data show that TCDD suppressed IgG1 expression and secretion in EAE and in response to LPS + IL-4 in vitro. Part of the pathophysiology of EAE involves production of pathogenic antibodies that can recruit cytolytic cells to destroy MOG-expressing cells that comprise myelin, so inhibition of IgG1 likely contributes to TCDD’s disease attenuation. Moreover, these data suggest that suppression of IgG1 might also account for TCDD’s ability to suppress other autoimmune disease models such as autoimmune type 1 diabetes [37], autoimmune uveoretinitis [38], and murine systemic lupus erythematosus [39]. These studies further suggest that less toxic AhR ligands that attenuate autoimmune disease could possess efficacy as immunosuppressants through suppression of IgG1.

## Data Availability

All data are available upon reasonable request.

## Supplementary Materials

The following supporting information can be downloaded at: https://www.mdpi.com/article/10.3390/antib11010004/s1. Figure S1: EAE clinical scores.
