# Supplementary figures and images for: TCDD Inhibition of IgG1 Production in Experimental Autoimmune Encephalomyelitis (EAE) and In Vitro
Source: Antibodies (Basel). 2022 Jan 9;11(1):4. doi: 10.3390/antib11010004 (PMC8788515; doi:10.3390/antib11010004)

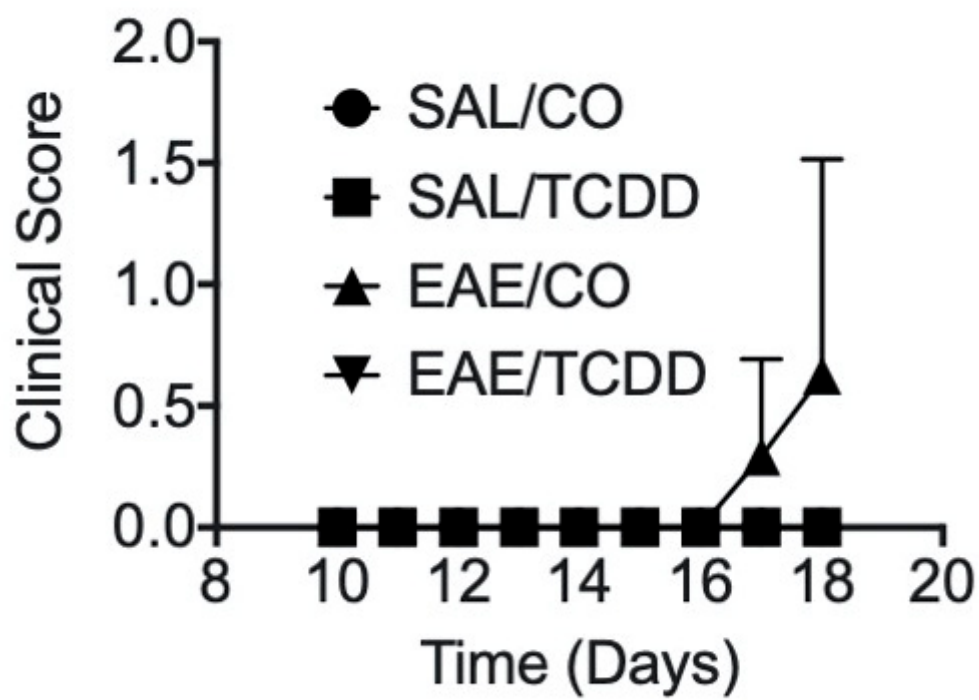

**Figure S1.** EAE clinical scores.

Supplement: Supplementary file 1 [file antibodies-11-00004-s001.zip › antibodies-1439069-supplementary.pdf]
